# Estimating COVID-19 exposure in a classroom setting: A comparison between mathematical and numerical models

**DOI:** 10.1063/5.0040755

**Published:** 2021-02-24

**Authors:** Aaron Foster, Michael Kinzel

**Affiliations:** University of Central Florida, Mechanical and Aerospace Engineering, Orlando, Florida 32766, USA

## Abstract

The COVID-19 pandemic has driven numerous studies of airborne-driven transmission risk primarily through two methods: Wells–Riley and computational fluid dynamics (CFD) models. This effort provides a detailed comparison of the two methods for a classroom scenario with masked habitants and various ventilation conditions. The results of the studies concluded that (1) the Wells–Riley model agrees with CFD results without forced ventilation (6% error); (2) for the forced ventilation cases, there was a significantly higher error (29% error); (3) ventilation with moderate filtration is shown to significantly reduce infection transmission probability in the context of a classroom scenario; (4) for both cases, there was a significant amount of variation in individual transmission route infection probabilities (up to 220%), local air patterns were the main contributor driving the variation, and the separation distance from infected to susceptible was the secondary contributor; (5) masks are shown to have benefits from interacting with the thermal plume created from natural convection induced from body heat, which pushes aerosols vertically away from adjacent students.

## I. INTRODUCTION

Currently, there are more than 62.2 × 10^6^ COVID-19 cases and nearly 1.5 × 10^6^ deaths due to COVID-19.^1^ During the pandemic, many school re-openings have occurred but, in general, remain well below full capacity due to the concerns associated with increased COVID-19 infections. The airborne transmission path has been evaluated^2,3^ and recently recognized by the World Health Organization^1^ (WHO) and the Center for Disease Control and Prevention^4^ as a mode of transmission of SARS-CoV-2. Improved insights and understanding of respiratory droplets describe a continuum that spans from larger droplets that travel distances less than 6 ft (∼2 m)^5,6^ along with smaller droplets that can travel much further in the buoyant gas clouds produced from exhalation and weak ambient currents.^7,8^

Prevention of airborne transmission of SARS-CoV-2 demands attention to the Heating, Ventilation, and Air Conditioning (HVAC) systems. For example, a recent study evaluated the 2020 Presidential debate^9^ including the effects of the HVAC system on transmission risk. HVAC recommendations include ensuring HVAC systems are in good working order and configure the systems such that they maximize fresh air within the limitations of the unit.^10,11^ The overall suggestions imply that the system will dilute aerosols with this fresh air to reduce the probability of transmission.

As the COVID-19 pandemic has progressed, there has been increasingly widespread use of calculators for estimating airborne exposure risk for indoor spaces. Most of these calculators are based on the Wells–Riley equation, which calculates the probability of infection from pathogens (such as SARS-CoV-2). These probabilities are a function of quanta (viruses released), exposure time, ventilation rate, room volume, and other factors.^12^ Outside of the calculators' practical use cases, such calculations are being used to mathematically compare the risk of typical indoor spaces.^13,14^ Other models have also been developed and studied for pathogen transmission.^15^

Wells–Riley and its derivatives are formulated on a basic model that is simplified through a number of assumptions that demand verification in real-world settings. With respect to classical fluid dynamics, Wells–Riley in the Gammaitoni and Nucci^16^ form can be described as combining the application of Reynolds transport theorem while including the probability of inhaling a given quantity of something (e.g., an infection dose of SARS-CoV-2). Consider the control volume as a room with an influx of quanta (with a specified concentration) from a host breathing, influx of clean air that dilutes, along with filtration (a sink of quanta) from the HVAC. The overall model provides temporal variation of quanta that can be linked to transmission probability. The Wells–Riley model relies on several key assumptions on airflow behavior. One assumption that is the transmission probability is highly sensitive to is that the quanta are uniform or completely mixed into the room. More recent implementations of the Wells–Riley calculation have included a “mixing factor” to represent incomplete mixing of air in a space. This mixing factor^17^ represents the efficiency that the ventilation system exchanges the complete volume of air in a space. A low mixing factor value implies that the room is well mixed without dead zones or short circuiting of flow; such factors are important as it enables HVAC systems to filter pathogens and particulate from all the air in the room, rather than a small portion. In general, this mixing efficiency assumption is a limitation of the Wells–Riley model, whereas Computational Fluid Dynamics (CFD) models capture many additional and complex effects and allow a much more detailed investigation in the result space compared to test methods. These CFD models have the potential to provide further insight into airborne diseases transmission routes and guide us on proper use of the more practical Wells–Riley models.

Previous studies have investigated the COVID-19 risk in similar spaces using CFD. These studies have illustrated how supply and return vent locations can create recirculation zones, which results in a higher exposure risk.^18,19^ CFD is a powerful tool that can be continued to be leveraged to understand which factors of a ventilation design can affect the risk of airborne disease transmission. By combining CFD modeling principles used in past studies with the principles of the Wells–Riley model for disease transmission, we can develop a more complete understanding of infection risk in a space. Additionally, we can use CFD studies to determine more appropriate mixing factors to be used in Wells–Riley calculations.

In the following, comparison studies of Wells–Riley and CFD are developed. This paper initiates with the development of the mathematical formulation of modern forms of Wells–Riley along with equivalent methods in the context of CFD. This paper then details the approach to compute probability of transmission through airborne transmission routes. We then develop a model classroom that is used to study the effectiveness of Wells–Riley-based models. This scenario is used to compare well ventilated and poorly ventilated rooms. The comparisons are then used to develop an understanding of the accuracy of Wells–Riley models.

## II. METHODS

In order to establish the methods, a summary of the mathematical and numerical models used in the present comparisons for pathogen exposure in the classroom setting is provided. The background of the Wells–Riley calculations is reviewed, and the more recent time-varying form by Gammaitoni and Nucci is presented for the comparison. Finally, the CFD model equations, computational domain and mesh, and assumptions are also described for completeness.

In the context of the present comparison studies, two scenarios are considered and are summarized in Table I. The first case considered a classroom with no ventilation; this could be an example of a moderate temperature day where the HVAC system is in low demand and does not run for the length of a class period, or this could also represent a baseboard or radiator type heating case where there is little or no mechanical ventilation occurring. The second scenario considers the effects of forced mechanical ventilation at a rate of 3.4 Air Changes per Hour (ACH). This represents the typical airflow into a classroom of this size from a short survey of recent public-school classroom plans. Both scenarios were analyzed with different levels of grid and time step refinement to confirm grid and temporal independence of the results.

**TABLE I.** Numerical and mathematical model cases.

Case	Description	Ventilation	Filtration	Flow time	Supply temp	Ambient temp
1	No ventilation	0.0 ACH	N/A	3600 s (1 h)	N/A	23.9 °C (75 °F)
2	Mechanical ventilation–heating	3.4 ACH	MERV^a^ 11 (72%)	3600 s (1 h)	32.2 °C (90 °F)	23.9 °C (75 °F)

^a^ Minimum Efficiency Reporting Values.

### A. Wells–Riley mathematical model for infection probability

Short class durations drive a need to study time-varying infection probability. Hence, for this study, we will use an adaption of Wells–Riley developed by Gammaitoni and Nucci, which considers the accumulation of particles as a time dependent calculation.^16^ This calculation is based on a simple source-sink model, and the concentration portion of the calculation can be derived with an ordinary differential equation based on the rate of a source (infected individual) and a sink (ventilation) in a closed volume. This updated form of the Wells–Riley calculation also introduces the volume of the space being considered.^16^

The model is given as follows:

$$P=1-e^{\left(-\frac{pIq}{V}\;\frac{Ct+e^{\left(-Ct\right)}-1}{C^{2}}\right)}.$$ (1)

This equation directly calculates *P*, which is the probability of infection (%). This probability relates to *I*, which is the number of infected persons (assumed to be one for this study), *p* is the pulmonary ventilation rate (m^3^/h) estimated to be^20^ 800 L/h, *q* is the quanta generation rate (h^−1^) estimated to be 100/h based on a previous study,^13^ *C* is the equivalent ventilation rate (h^−1^), *V* is volume of the space (m^3^), and *t* is time (s). In the context of this equation, it is important to understand how it relates to a transmission event. According to Wells, transmission occurs 63% of the time (based on a Poisson distribution) when one inhales a quantum of the pathogen, which can also be described as an infection dose of viral particles inhaled.^21^ Hence, the generation rate is the rate of quanta exhaled from the ill host. This initial form can be adapted to account for various other important mechanisms.

As previously mentioned, the equivalent ventilation rate demands adaptions to account for mixing and other mechanisms. The form used in this work is given as

$$C=\frac{Q}{K}\frac{\eta_{filter}}{V}+k_{deposition}.$$ (2)

Here, *Q* is the ventilation rate (m^3^/h), *K* is the mixing factor, *η*_*filter*_ is the equivalent filtering efficiency for the HVAC system, which accounts for removal of respiratory droplet nuclei in the HVAC filter,^22^ and *k*_*deposition*_ is the deposition rate (h^−1^). The value of *K* normally used is 1, which implies perfect mixing; normally, a mixing factor will vary from ideal mixing (*K* = 1) to poor mixing^17^ (*K* > 10). Finally, *k*_*deposition*_ refers to quanta that deposition surfaces due to settling. As our focus is on small aerosols (<10 *µ*m) in well mixed rooms, this value is negligible. In the context of these assumptions, *C* can be thought of as the percentage of the room volume that the ventilation system exchanges with clean air per hour.

As many schools have mask mandates during the COVID-19 pandemic, it is important to consider such a scenario. In this work, we consider mask filtration, which is assumed to be based on a surgical mask with a gap fit that yields a filtration efficiency (*η*_*mask*_) of^23^ 44%. Filtration is incorporated into the Wells–Riley calculation by assuming that both the quanta and the inhaled air are filtered at the same rate, which considered together becomes a factor of ${\left(1-\eta_{mask}\right)}^{2}$,

$$P\left(t\right)=1-e^{\left(-\frac{pIq{\left(1-\eta_{mask}\right)}^{2}}{V}\frac{Ct+e^{\left(-Ct\right)}-1}{C^{2}}\right)}.$$ (3)

The overall adaptation of the Wells–Riley model, hence, incorporates effects of mixing, HVAC and its filtration, and filtration from masks.

### B. Numerical methods for infection probability

The numerical model was solved using a commercial CFD code (Star-CCM+), which utilizes the finite volume method for discretization of the fluid flow equations that satisfy mass, momentum, and energy conservation. In this context, an incompressible-ideal gas model is used along with energy balance equations to capture buoyancy effects of the warm exhaled air and the thermal plume effect around the bodies of the occupants. The conservation of air mass (in Cartesian tensor notation) is given as

$$\frac{\partial \rho }{\partial t}+\frac{\partial \rho V_{i}}{\partial x_{i}}=0.$$ (4)

In the context of this formulation, only one gas species is assumed. Additionally, *ρ* is the gas density, t is time, and *V*_*i*_ is the velocity in the *i*-direction. The momentum equations in the context of a hybrid Reynolds Averaged Navier Stokes (RANS)/Large Eddy Simulation (LES) are solved. Specifically, a Detached Eddy Simulation (DES)^24^ was chosen to simulate aerosol dispersion in the context of turbulent mixing. This model utilized a Spalart–Allmaras single equation^25^ turbulence model in the DES model to calculate the unresolved turbulence. Away from the walls, the Improved Delayed Detached Eddy Simulation (IDDES) model captures large-scale eddies and turbulent mixing; this model is similar to a LES model that captures these large scales by solving filtered governing equations (incompressible Navier–Stokes) directly rather than with an averaged velocity term in RANS models. The momentum equations are solved as follows:

$$\frac{\partial \left(\rho V_{j}\right)}{\partial t}+\frac{\partial \left(\rho V_{i}V_{j}\right)}{\partial x_{i}}=-\frac{\partial p_{g}}{\partial x_{j}}+\rho g_{j}+\frac{\partial {\bar{\bar{\tau }}}_{ij}}{\partial x_{i}}.$$ (5)

Here, *p*_*g*_ is the gauge pressure in the fluid, and *g*_*j*_ is the gravity vector. Additionally, the ${\bar{\bar{\tau }}}_{ij}$ term is the viscous-stress tensor, which can be expressed as

$${\bar{\bar{\tau }}}_{ij}=\mu_{m}\left(\frac{\partial V_{i}}{\partial x_{j}}+\frac{\partial V_{j}}{\partial x_{i}}\right)+\left(\lambda_{q}-\frac{2}{3}\mu_{m}\right)\frac{\partial V_{l}}{\partial x_{l}}\delta_{ij},$$ (6)

where *μ*_*m*_ is the modeled viscosity, which is defined as the sum of the modeled turbulence viscosity provided by the IDDES model and the viscosity of the gas (1.855 × 10^−5^ Pa s). The energy equation, used to capture thermal plumes and buoyant effects in the context of the HVAC, is provided as follows:

$$\frac{\partial \left(\rho e\right)}{\partial t}+\frac{\partial \left(\rho eV_{i}\right)}{\partial x_{i}}=-\frac{\partial \left(p_{f}V_{i}\right)}{\partial x_{i}}+\frac{\partial V_{i}{\bar{\bar{\tau }}}_{l,ij}}{\partial x_{i}}+\frac{\partial }{\partial x_{i}}\left(k\frac{\partial T}{\partial x_{i}}\right),$$ (7)

where *e* is the internal energy of the gas ($e=c_{v}T\left.\right)$, *k* is the thermal conductivity, and T is temperature. The state equation couples energy to the density through

$$\rho =\frac{p_{0}}{RT},$$ (8)

where T fluctuates, *p*_0_ is the ambient pressure (101 000 Pa), and R is 287 J/kg/K. Finally, in order to track the exhaled quanta in the context of dispersion through the room and the HVAC system, a quanta dispersion equation (QDE) is solved as a one-way coupled convection equation that represents the exhaled quanta that contain viral particles. Others have shown that the small dehydrated respiratory droplets (less 2 *μ*m) will follow the airflow and the QDE can represent the droplets rather than a more computationally expensive discrete particle model (DPM).^26^ This equation is given as follows:

$$\frac{\partial \rho Q_{C}}{\partial t}+\frac{\partial \rho V_{i}Q_{C}}{\partial x_{i}}=0,$$ (9)

where *Q*_*C*_ is the quanta concentration (i.e., *quanta*/m^3^).

In the context of these equations, second order numerical discretization schemes (temporal and spatial) are utilized along with a Semi Implicit Method for Pressure Linked Equations (SIMPLE) segregated solver. This work used mesh generated in Star-CCM+ containing 1.9 × 10^6^ cells with a time step size of 0.5 s. These discretization parameters guided final results based on a series of mesh and time step size studies, indicating that they sufficiently resolve the dynamics of interest.

#### 1. Calculating infection probability

**FIG. 1.**
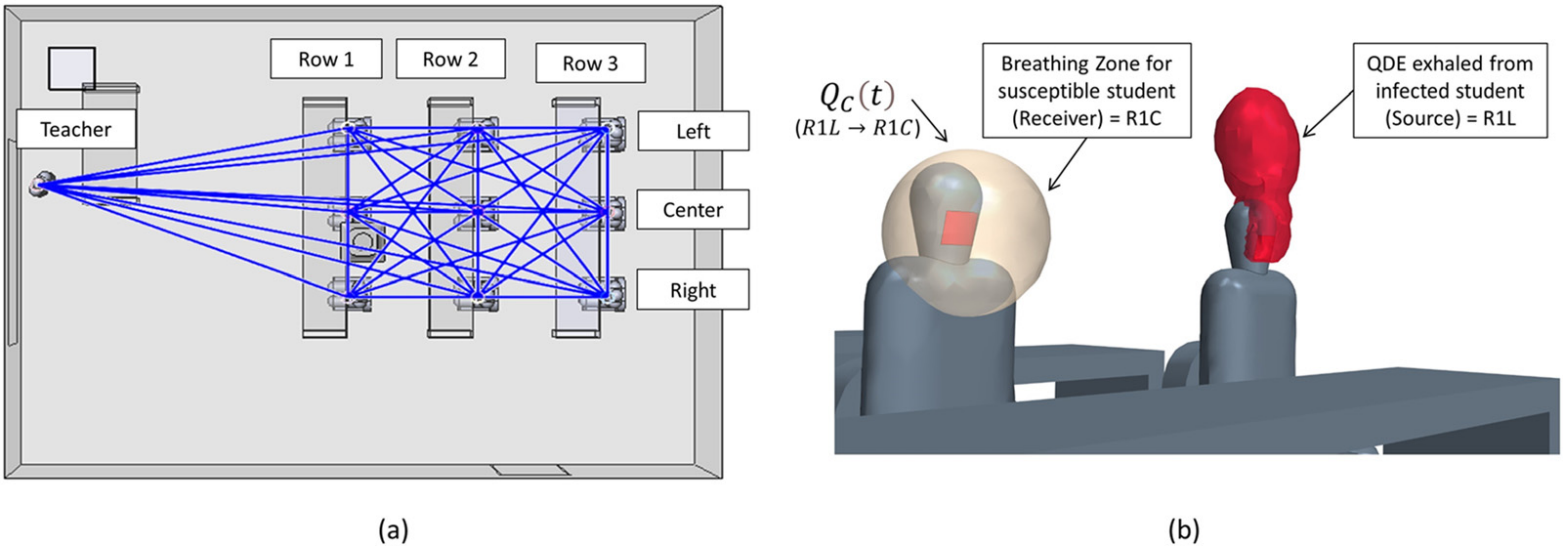
CFD representation of transmission routes: (a) potential transmission routes and (b) breathing zone and QDE for one transmission route.

To compare the CFD results to the Wells–Riley equation, the infection probability in each breathing zone is demanded. In the context of groups, it is important to consider all occupants as potentially being infectious. An infection-susceptibility model can be extracted from a CFD simulation to investigate all possible transmission routes and determine risk depending on both location and distance from an infected individual. An example of this network for the present classroom study is depicted in Fig. 1(a). Any space having a total of n persons has n − 1 potential other routes that could infect that person, leading to a total number of transmission routes of *n*(*n* − 1). In the context of the classroom, this leads to 10(9) or 90 transmission paths. Using such a detailed model allows us to better understand “super-spreading” events, infection probability, and the relationship of transmission with respect to the distance from an infected individual. For each transmission route, the QDE from a single source is tracked at the location of a susceptible occupant breathing zone. It is also possible to directly track the quanta inhaled; however, the concept of a “breathing zone” allows for all transmission routes to be calculated simultaneously, which leads to more efficient calculations. Comparison studies for this model have shown the breathing zone concept to closely represent the actual inhaled quanta.

In the context of the breathing zone, the concentration inhaled for a susceptible person can be evaluated. A direct evaluation can be developed through the temporal integration of the product of an assumed breathing volume and the breathing zone concentration. The result provides the total volume inhaled, which can then be used to calculate the infection probability from the CFD analysis.

Transmission implies that one inhales an infection dose of quanta exhaled from an infection person. We define this infectious dosage in terms of a quanta volume, *V*_*quanta*_(m^3^), which is defined as

$$V_{quanta}=\frac{p}{q}=\frac{700\frac{L}{hr}}{100\frac{1}{hr}}=0.007m^{3}.$$ (10)

Recall that *p* is the pulmonary ventilation rate, which is equal to 700 L/h for talking,^20^ and *q* is the quanta generation rate, which is estimated as an average value of 100 h^−1^ from the work of Buonanno *et al.* for a resting activity level. Note that these values do not change for the different scenarios studied and this can be calculated as a constant value. From the CFD analysis, the exhaled concentration is directly computed and varies as a function of time for each breathing zone. With these data, we can calculate the inhaled volume over time in

$$V_{inhaled}\left(t\right)=\int_{0}^{t}pQ_{C}\left(t\right){\left(1-\eta_{mask}\right)}^{2}dt,$$ (11)

where *p* is the pulmonary ventilation rate (m^3^/h), $Q_{C}\left(t\right)$ is the CFD concentration over time, and *η*_*mask*_ is the mask filtering efficiency (%). Note that in this study, $Q_{C}\left(t\right)$ is the concentration for an assumed single infected person in the classroom. This concentration could be used to represent any number of infected individuals in the space by summing together multiple passive scalars from multiple infected individuals. For this study, the current prevalence rates suggest that it is unlikely that there would be multiple infected students in a small classroom but would be a relevant comparison for larger venues.

From the quanta volume and inhaled volume, we can calculate the fraction of an infectious dose and use the same Poisson's distribution to make the result equivalent to the typical form of the Wells–Riley calculation,

$$P\left(t\right)=1-e^{\left(-\frac{V_{inhaled}\left(t\right)}{V_{quanta}}\right)},$$ (12)

where *P* is the probability of infection (%) for a given duration of time (t).

#### 2. Geometry

**FIG. 2.**
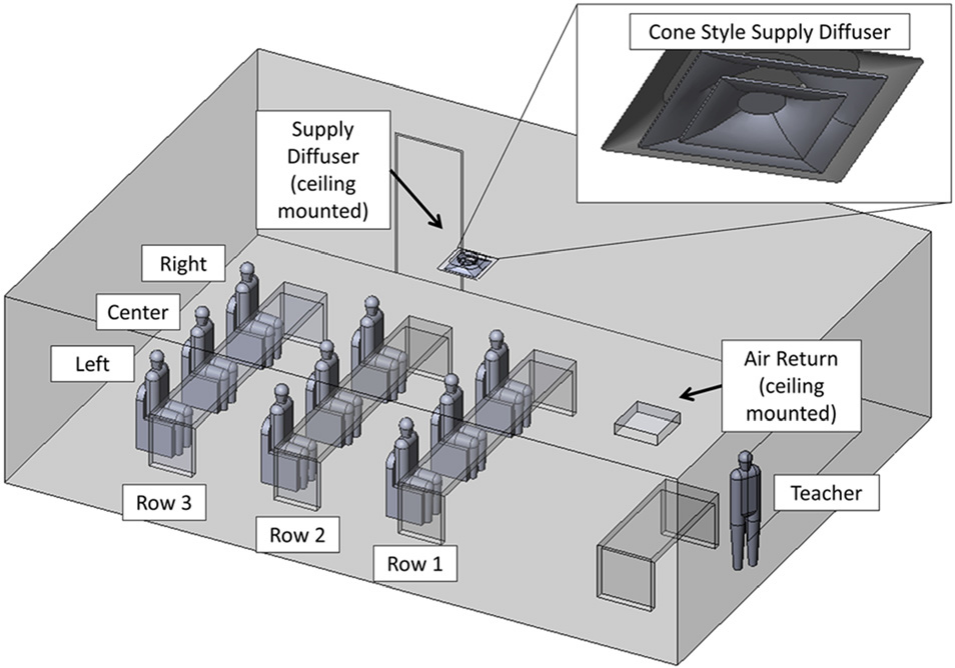
CAD representation of a classroom.

**FIG. 3.**
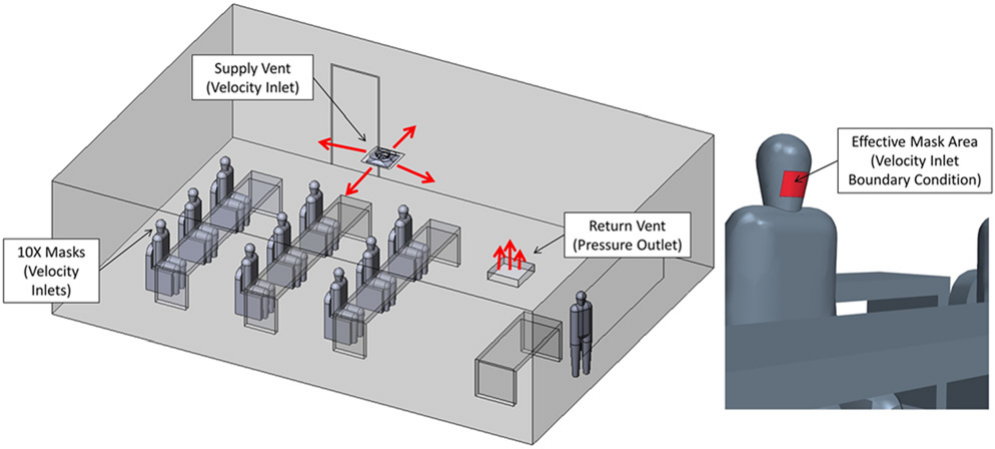
Description of boundary conditions.

The classroom configuration including the naming schema of the students and teacher is shown below in Fig. 2. The classroom has a floor area of 66 m^2^, which is based on rectangular dimensions of 9.5 m long and 7.0 m wide. The height of the classroom is 2.7 m. On the ceiling of this classroom is a single, centrally located inlet supply along with a single, corner-located return supply. The inlet supply is modeled after a common commercial, cone-style supply (Titus TMS-AA) with a 0.5 m diameter inlet section. The return vent is a 0.6 m square section, which is recessed slightly above the ceiling plane. In the context of this classroom, these HVAC ducts drive a flow consistent with a standard room with commercial-grade venting (Fig. 3).

#### 3. Boundary conditions

The boundary conditions are summarized in Table II below. The thermal settings of the room were selected as isothermal walls to maintain a constant ambient temperature for the duration of the CFD analysis. In addition to the boundary conditions listed below, there was also a special treatment of *Q*_*C*_ exiting through the air return, which is then recirculated through the supply duct. Many real classroom air handlers will typically supply several spaces; because of this, the aerosols exhaled by occupants are distributed to other spaces, which further dilutes the amount being recirculated back into a given space. For this study, it was assumed that all the air leaving the space through the return vent is recirculated back to the supply duct and travels through an air filter in the air handler unit. It was assumed that a MERV 11 (Minimum Efficiency Reporting Values) filter was being used, which has a droplet nuclei weighted filtering efficiency (*η*_*filter*_) of 72% according to a NAFA report.^22^ Hence, the filtering needed to be accounted for in the CFD model through the custom function that filters the aerosols passing through the supply vent for each of the ten passive scalars (representing nine students and one teacher). This is performed using the mass-averaged quanta concentration computed at the return vent, computed as

$${\left.\bar{Q_{C}\left(t\right)}\right|}_{RetVent}=\frac{\int_{RetVent}\rho Q_{c}\left(t\right)V_{i}ds_{i}}{\int_{RetVent}\rho V_{i}ds_{i}},$$ (13)

which is returned into the supply vent decremented by 1 − *η*_*filter*_. In summary, these boundary conditions represent a school room with ten persons exhaling, in the context of various HVAC conditions with recycled air that is also filtered.

**TABLE II.** Summary of boundary conditions.

Description	Type	Flow	Pressure	Temperature	Quant concentration
Masks	Velocity inlet	$3.2\times 10^{-4}\frac{m^{3}}{s}$ (0.67 cfm)	N/A	35 °C (95 °F)	100 quanta/h
Supply vent	Velocity inlet	$0.17\frac{m^{3}}{s}$ (360 cfm)	N/A	Case 1: N/ACase 2: 32.2 °C (90 °F)	Case 1: N/ACase 2: $\left(1-0.72\right){\left.\bar{Q_{C}\left(t\right)}\right|}_{RetVent}$
Return vent	Pressure outlet	N/A	*p*_0_	N/A	N/A
Walls and ceiling	Wall	N/A	N/A	Case 1: 23.9 °C (75 °F) Case 2: 21.1 °C (70 °F)	N/A
Bodies	Wall	N/A	N/A	29.4 °C (85 °F)	N/A
Heads	Wall	N/A	N/A	33.3 °C (92 °F)	N/A
Air initial condition	Fluid	N/A	N/A	23.9 °C (75 °F)	N/A

To represent current conditions in schools where masks are mandated (common practice in the pandemic), a simplified representation of masks as a boundary condition is considered. Abkarian *et al.* demonstrated that talking without a mask produces “puff-like” flow and has an influence on the mixing of air, which could produce more favorable results in this study due to increased mixing of the air.^27^ The pulmonary flowrate *p* (m^3^/s) and the effective mask area *A*_*mask*_ (m^2^) are used to calculate the mask outlet velocity. The assumed mask area is 82 cm^2^ and approximated the actual effective area of a mask. The exhaled flow behavior observed in the CFD model results agrees with a study where schlieren imaging is utilized to demonstrate how the exhaled air out of the mask is slow and follows the thermal plume vertically rather than being projected forward,^28^

$$v_{mask}=\frac{p}{A_{mask}}.$$ (14)

## III. RESULTS AND DISCUSSION

### A. Case 1: No ventilation

**FIG. 4.**
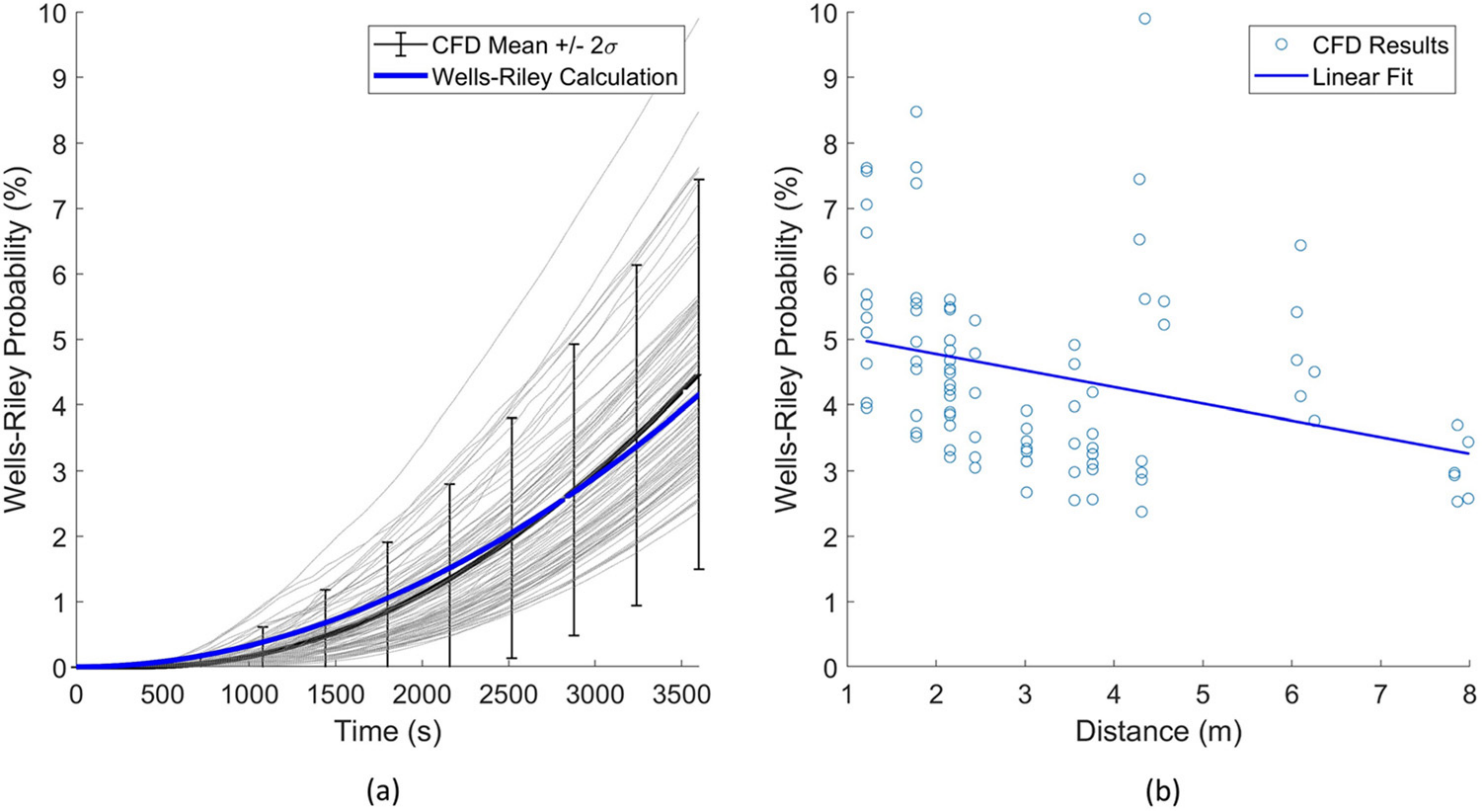
Case 1 results: (a) infection probability vs time and (b) infection probability vs distance.

The first case evaluated considers the room in an unventilated state, which implies that the filters are not functioning and the HVAC does not drive flow throughout the classroom. The scenario represents a classroom without HVAC, fans, and the windows closed. A summary of the results for this case are provided in Table III. From these results, the mean infection probability from the numerical model agreed well with the Wells–Riley mathematical model with a relative error of 6%. Recall that the CFD model can elucidate 90 transmission routes. These 90 transmission routes are displayed, in comparison to the Wells–Riley prediction. Figure 4(a) indicates predictions of the probability of infection as a function of time, which increases due to the increasing probability of inhaling a quanta from an infection person. Figure 4(b) indicates the overall probability after 3600 s. The statistics of these 3600 s data are also indicated in Table III. In general, Wells–Riley agrees with the mean CFD model prediction (∼6% underprediction) for the time duration considered.

**TABLE III.** Case 1 result summary.

Calculation method	Ventilation rate	Mixing factor	Mean probability	Maximum probability	+/−2σ Std. deviation
Wells–Riley	0 ACH	K = 1	4.2%	N/A	N/A
CFD	0 ACH	N/A	4.5%	9.9%	+/−3.0%
		Relative error	6%		

Additional evaluation of Fig. 4 indicates two important findings. The general trend indicated in Fig. 4(b) is the CFD prediction of infection probability after 3600 s and the distance from the infected host. The prediction has a coplot of linear regression of this trend. The detailed CFD analysis indicated wide fluctuations in infection risk for the different persons and a relatively weak correlation of risk reduction with increased distance. One particular observation is that the highest probability of transmission occurs at 4.5 m (15 ft), which is further than physical-distancing guidelines of 2 m (6 ft). Additionally, at 6 m (20 ft), there are scenarios with similar infection probabilities. This highlights that for an airborne pathogen, mask mandates, number of occupants, and proper HVAC operation drive protection from airborne pathogens, while physical distancing, on the other hand, has only a minor effect of reducing transmission. Additionally, referring back to Table III, in order to capture these fluctuating probabilities within two standard deviations, the mean CFD prediction (similar to the Wells–Riley prediction) demands roughly 66% of the mean. These effects are attributed due to local air patterns that affect aerosol concentration dynamics within the classroom. These detailed fluid dynamics are not considered in the Wells–Riley model and can lead to nonconservative estimations of infection probability, which, for this scenario, may be reasonably accounted for with a 66% inflation factor to increase the conservative nature of the prediction.

**FIG. 5.**
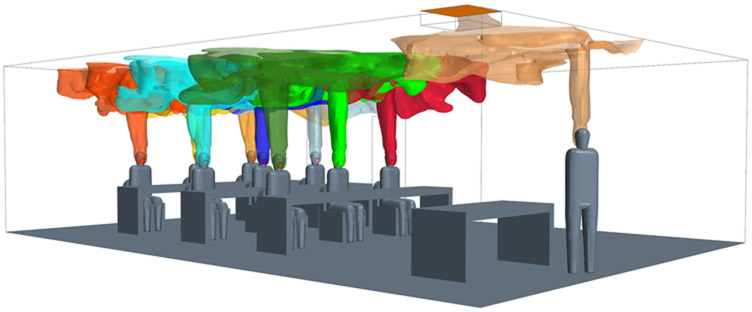
Initial thermal plumes and stratification created from the exhaled air at t = 35 s.

In this unventilated room, the mechanisms creating significant air movement are driven by buoyancy from the exhaled air as well as thermal plumes created from the heated body. The effect is clearly indicated at *t* = 35 s in Fig. 5. Here, the plume of each person is indicated with an isosurface of the *Q*_*C*_, which indicates that an upward advection driven by thermal buoyancy. The Wells–Riley calculation assumes complete mixing leading to a single prediction. Alternatively, the CFD results capture actual aerosol concentrations driven by these thermal currents that lead to a range of results (2.4%–9.9%) that are not captured with the mathematical model.

Reflecting these results back to application of the Wells–Riley model, there are some key points. One clear scenario where Wells–Riley demands usage with caution is in the forensic investigations of super-spreading events. In such cases, Wells–Riley calculations appear to underpredict the higher infection probability of position due to developing air patterns with respect to infected individuals. Additionally, in risk assessment use, the mean value of all transmission routes is considered to represent average risk for comparison to the Wells–Riley calculation. One could consider the worst-case transmission route; however, this is likely overly conservative. Statistical representations, such as those provided in these predictions, may provide guidance on transmission risk reduction for the bulk of the persons in the working environment. Additionally, it is interesting to note that the mean infection probability decreases with the distance between the occupants [Fig. 4(b)], which appears to capture the bulk of the transmission paths. However, the large scatter of isolated events leads to a peak infection probability at a distance of 4.5 m. Such interactions demand additional attention to the detailed flow physics.

### B. Case 2: Mechanical ventilation

**FIG. 6.**
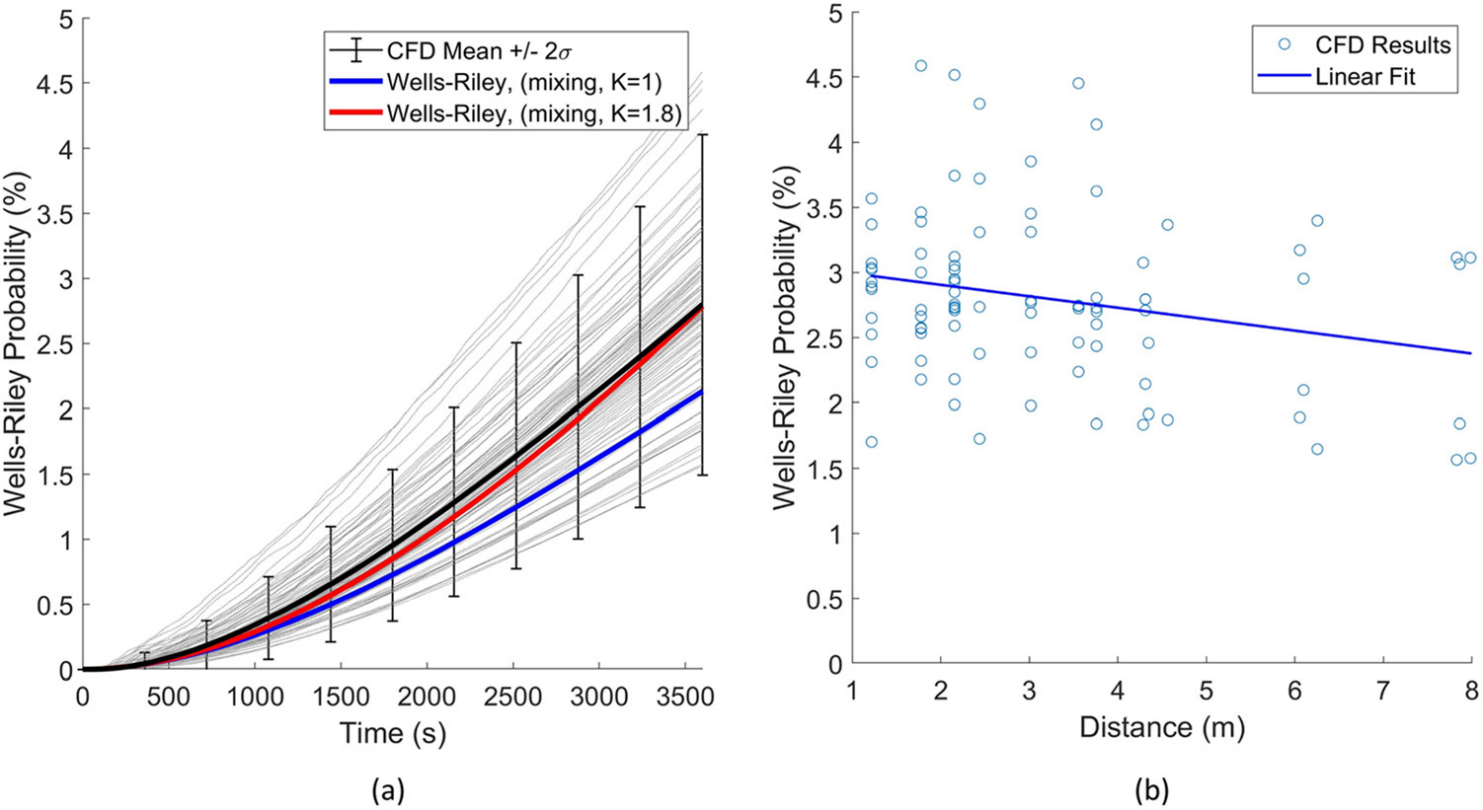
Case 2 results: (a) infection probability vs time and (b) infection probability vs distance.

The second case considers the room with the addition of forced ventilation through a ceiling supply vent; this case also adds filtration and represents a portion of the *Q*_*C*_ returning through the supply vent based on the amount escaping the return vent and the filtration rate. A summary of these results is provided in Table IV. From these results, there was less agreement with the Wells–Riley mathematical model with the ideal mixing assumption than the first case with a relative error of 29%. As previously discussed for case 1, 90 transmission routes are also displayed, in comparison to the Wells–Riley prediction. Figure 6(a) indicates predictions of the probability of infection as a function of time, which increases due to increasing the probability of inhaling a quanta from an infection person. Note that a corrected mixing factor was also calculated (*K* = 1.8) in the Wells–Riley method to reach the same average probability as the CFD results. Note that this factor accounts for both the ventilation mixing and the air pattern exposure, which is unique to the CFD approach. Figure 6(b) indicates the overall probability after 3600 s. The statistics of these 3600 s data are also indicated in Table IV. In general, Wells–Riley provided a significantly lower infection probability than the mean CFD model prediction (29% underprediction).

**TABLE IV.** Case 2 result summary.

Calculation method	Ventilation rate	Mixing factor	Mean probability	Maximum probability	+/−2σ Std. deviation
Wells–Riley	3.4 ACH	K = 1	2.1%	N/A	N/A
CFD	3.4 ACH	N/A	2.8%	4.6%	+/−1.3%
		Relative error	29%		

Considering the effect of distance on the infection probability, a similar trend is seen in Fig. 6(b) as the first case. The linear fit of the results shows that, overall, there is less of an influence on the distance with added ventilation; this further supports the idea that the distance is not a main indicator of infection probability for indoor spaces. It is seen, however, that the results follow more of a distinct trend, and the highest probability is at a shorter distance of 2 m (6 ft) and the lowest probability is at the furthest distance of 8 m (27 ft).

Considering the effect of the forced ventilation on these results in Fig. 6(a) reveals several interesting findings when compared to the no-ventilation case. The reduced variation in the individual transmission can be clearly seen; this is observed to be due to steady flow patterns emerging with the forced ventilation. The no-ventilation case had a very weak source for mixing from buoyant flow; in comparison, the forced flow creates much stronger advection, which results in more stable air patterns. Another observation is that the average infection probability is much lower with the ideal mixing assumption. Although the forced ventilation improved the consistency of the air patterns in the room, which resulted in less variation overall, the average is much higher than the Wells–Riley calculation result. This indicates that stable recirculation zones are affecting the flow in areas of the room; in the CFD model, this is seen in areas near the walls where the laminar ceiling flow becomes detached and creates eddies. The eddies trap aerosols due to the decaying air velocity and circulating flow, these effects allow *Q*_*C*_ to build concentration and result in higher exposure, especially in the end of the room opposite the air return where the mixing factor is higher (poor mixing).

**FIG. 7.**
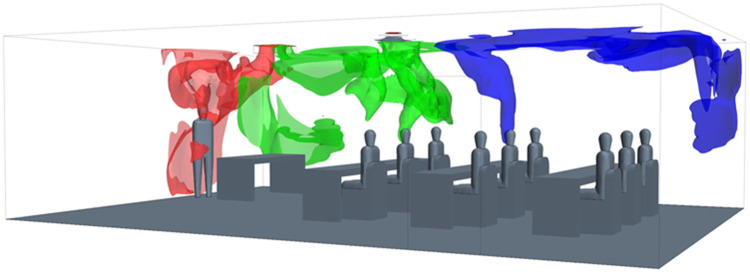
Initial air patterns created from the exhaled air at t = 35 s (only teacher, R1C, and R2C shown for clarity).

The mechanisms creating air movement are significantly different than the first case with the addition of ventilation. Rather than the flow being directed primarily from buoyancy driven air currents, the flow is now shown to be influenced mainly by the flow pattern from the ceiling mounted supply and return duct. This effect can be seen for *t* = 35 s in Fig. 7. The upward advection driven flow is seen again and similar to the first case, but it quickly becomes influenced by the ventilation flow air patterns from the supply vent flow, which creates an attached flow to the ceiling. The paths that the exhaled air takes are strongly influenced by the position in the room relative to the supply vent; this is seen to determine the corner of the room where the high concentration *Q*_*C*_ is directed. In Fig. 7, the R2C flow is shown to be directed to the rear of the room, and R1C is directed to the front of the room. The teacher is located near the return vent, which is seen to be an advantageous location for reducing *Q*_*C*_, which others in the room are exposed to. The Wells–Riley calculation assumes complete mixing leading to a single prediction. Alternatively, the CFD results capture actual aerosol concentrations driven by these thermal currents that lead to a range of results (1.6%–4.6%) that are not captured with the mathematical model.

These results again reinforce the same key points identified for the first case. In addition to these points, with ventilation, there is a stronger need to determine an appropriate mixing value for the space to achieve a better correlation between the Wells–Riley calculation and the average CFD results. There is an important distinction here between the first case with no ventilation, which does not utilize the mixing factor in the Wells–Riley equation due to the lack of a flow source into the space. This implies that Wells–Riley calculations for similar spaces with little or no ventilation will tend to agree better with the detailed CFD results than cases with higher ventilation and ideal mixing. Similar to case 1, we note that the mean infection probability decreases with the distance between the occupants [Fig. 6(b)], which appears to capture the bulk of the transmission paths. It is of note that the distance vs infection probability correlation is even weaker with the increased ventilation. However, the scatter is reduced and does follow a more consistent trend than the no ventilation case.

**FIG. 8.**
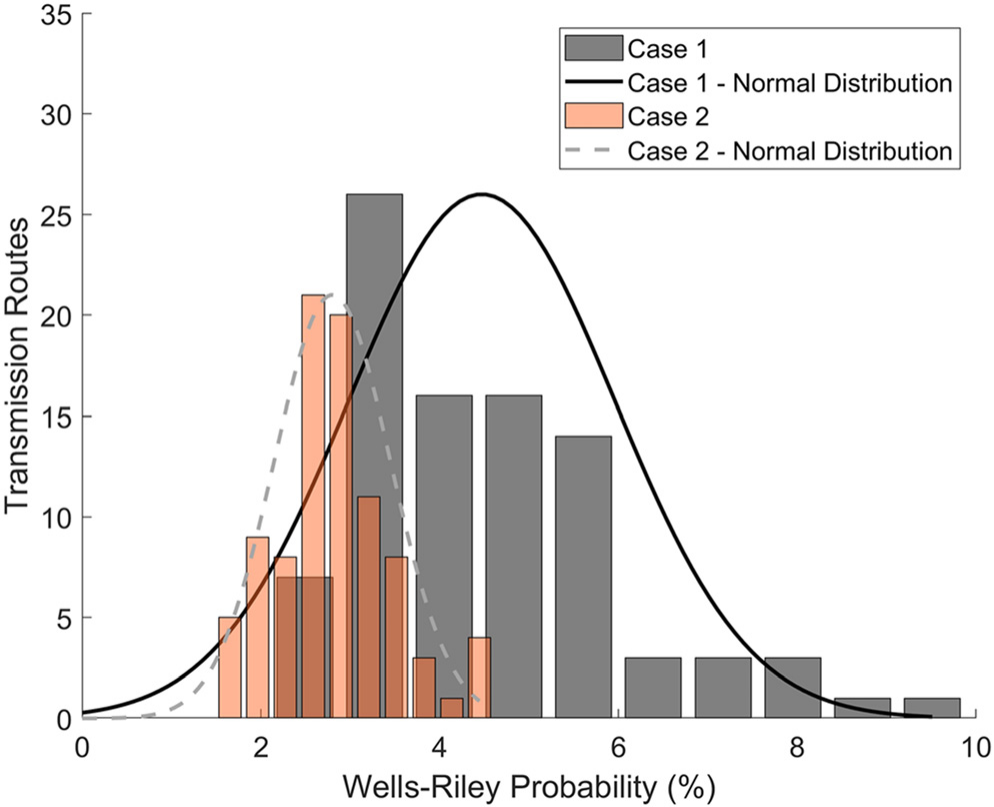
Distribution of transmission routes vs Wells–Riley probability for both cases at t = 3600 s.

The infection probability distribution of the 90 transmission routes is shown below in Fig. 8 as a comparison between case 1 and case 2 at t = 3600 s. A normal distribution is shown for each case and can be used to understand how each of the infection probabilities varies as a function of transmission route probability. Case 1, for example, can be seen to have a slight bias toward the lower infection probabilities, while case 2 has a more even distribution and is a better fit with a normal distribution. This distribution of transmission routes could be extended to a modified Wells–Riley calculation to better understand infection risks in a more probabilistic manner. Additionally, since there is a base concentration that builds in the space, which can be seen in the lower results in Fig. 8, a skewed distribution could be used for scenarios where the infection probability is increased.

These results further emphasize the need for airborne transmission models to consider the detailed flow physics, and there is a need to understand the distribution of infection probabilities of the potential transmission routes.

## IV. DISCUSSION AND RECOMMENDATIONS

From this comparison of the Wells–Riley infection probability calculation to the more detailed CFD model, several important effects were discovered, which should be considered when using tools such as spreadsheets and calculators based on the Wells–Riley model. As shown below in Table V, for case 1, the standard Wells–Riley calculation had good agreement on average with the CFD results for the classroom scenario in this study. There was a larger error for the mechanical ventilation case; this was considerable and should be considered in both risk evaluations and forensics work. For both cases, there was a wide range of results when considering individual transmission paths; this may be an important consideration depending on risk tolerance of the habitants. Future work is needed to further explore the effects of proximity, air patterns, room geometry, and ventilation parameters on the Wells–Riley results to understand the limitations of the mathematical model and to develop correction techniques to further improve the results of these calculations.

**TABLE V.** Result summary.

	Case	Ventilation	Filtration	Flow time	CFD results (+/−2σ) (%)	Wells–Riley results (ideal mixing) (%)
1	No ventilation	0.0 ACH	N/A	3600 s (1 h)	4.5 +/−3.0	4.2
2	Mechanical ventilation	3.4 ACH	MERV 11 (72%)		2.8 +/−1.3	2.1

Limitations of these modeling approaches should also be discussed. A view is emerging from the limited results presented that various factors such as boundary condition assumptions, temperatures, room layout, and ventilation type will have a large influence on the resulting infection probabilities due to their influence on the flow physics. There is a need to study these effects to determine which have the greatest effect on the results and what can be expected for different types of spaces. Additional effects, such as mixing from human movement in the space,^29^ will likely add to this variation. Considering further stochastic effects of SARS-CoV-2, humidity is believed to play an important role in the viability of the virus;^30,31^ for these scenarios, low indoor humidity was assumed and more detailed effects due to humidity and evaporation have not been explored. Finally, the roles of fomite and large droplet transmission are also not considered in this model and add to the complex nature of determining overall infection probability of indoor spaces.

## V. CONCLUSIONS

The infection probability in a typical classroom scenario was calculated using both mathematical (Wells–Riley) and numerical (CFD) methods. The Wells–Riley calculation had good agreement of infection probability when compared to the mean of the non-ventilated CFD model with a relative error of 6%. The overall range of infection probabilities was significant though and may be a contributor to the lack of consistency in where COVID-19 infections spatially occur in super-spreading events with poor ventilation present.

For the mechanical ventilation case, the Wells–Riley calculations under-predict risk compared to average results of the more detailed CFD models with a relative error of 29%. This case also had a wide range of results, but the variation was significantly less than the non-ventilated case. Ventilation in combination with filtration is shown to reduce the infection risk significantly in both the mathematical (50% reduction) and numerical (40% reduction) models even though only a moderate filter type (MERV 11) was assumed in the calculations.

The local air patterns created a much larger range of infection risk compared to the changes in risk from the source–receiver separation distance in the model. The risk from airborne SARS-CoV-2 exposure does not appear to be strongly correlated with the distance, and many of the peak exposures were observed outside of physical-distancing guidelines. This indicates that mask mandates, well designed HVAC systems, and the combination of exposure time with number of occupants are of increased importance compared to physical distancing. Such procedures are particularly important in the context of the newer, so called “super strains” of SARS-CoV-2 that are possibly more contagious through airborne routes. In the context of continuing operation, this work establishes the validity of the application of Wells–Riley calculations for airborne risk reduction in indoor spaces. Although the Wells–Riley mathematical model does not accurately capture the peak transmission events, it serves as a tool to estimate and rank the relative performance of various mitigations methods (these are typically exposure time, ventilation rate, filtration, and room size). These conclusions should provide a better understanding with Wells–Riley-based calculators when used to assess the infection risk of similar indoor spaces.

## AUTHORS' CONTRIBUTIONS

A.F. developed the model, concept, analysis, and developed the paper. M.K. supported and advised the modeling and analysis as well supported paper revisions.

## Data Availability

The data that support the findings of this study are available within the article.
